# Preoperative fasting times in elective surgical patients at a referral Hospital in Botswana

**DOI:** 10.11604/pamj.2016.23.102.8863

**Published:** 2016-03-16

**Authors:** Worknehe Agegnehu Abebe, Ambrose Rukewe, Negussie Alula Bekele, Moeng Stoffel, Mompelegi Nicoh Dichabeng, Jemal Zeberga Shifa

**Affiliations:** 1 University of Botswana, University of Botswana, Department of Anesthesia & Critical Care, Gaborone, Botswana; 2 University of Botswana, Department of Statistics, Botswana; 3 University of Botswana, Department of Emergency Medicine, Gaborone, Botswana; 4 University of Botswana, Department of Surgery, Gaborone, Botswana

**Keywords:** Preoperative fasting, fasting instructions, anaesthesia, elective surgical patient

## Abstract

**Introduction:**

Adults and children are required to fast before anaesthesia to reduce the risk of regurgitation and aspiration of gastric contents. However, prolonged periods of fasting are unnecessary and may cause complications. This study was conducted to evaluate preoperative fasting period in our centre and compare it with the ASA recommendations and factors that influence fasting periods.

**Methods:**

This is a cross-sectional study of preoperative fasting times among elective surgical patients. A total numbers of 260 patients were interviewed as they arrived at the reception area of operating theatre using questionnaire.

**Results:**

Majority of patients (98.1%) were instructed to fast from midnight. Fifteen patients (5.8%) reported that they were told the importance of preoperative fasting. The mean fasting period were 15.9±2.5 h (range 12.0-25.3 h) for solids and 15.3±2.3 h (range 12.0-22.0 h) for liquids. The mean duration of fasting was significantly longer for patients operated after midday compared to those operated before midday, p<0.001.

**Conclusion:**

The mean fasting periods were 7.65 times longer for clear liquid and 2.5 times for solids than the ASA guidelines. It is imperative that the Hospital should establish Preoperative fasting policies and teach the staff who should ensure compliance with guidelines.

## Introduction

American Society of Anesthesiologists (ASA) defined preoperative fasting as a prescribed period of time before a procedure when patients are not allowed the oral intake of liquids or solids [1]. Both adult and children are required to fast before anaesthesia with the goal of reducing the volume and acidity of the stomach content so as to reduce the risk of regurgitation and aspiration of gastric contents during the procedure. American Society of Anesthesiologists (ASA) developed guidelines that support more liberal preoperative fasting protocol. The summary of the fasting recommendation is as follow: the minimum fasting period of 2 hours for clear liquids, 4 hours for breast milk, 6 hours for solids, infant formula, nonhuman milk and light meal. The fasting period noted above apply to patients of all ages and undergoing elective procedures. Meals that include fried or fatty foods or meat may prolong gastric emptying time. An additional fasting time (8 hours or more) may be needed in these cases [1]. It has been reported that prolonged periods of fasting are unnecessary and may cause complications such as distress, confusion, instability, hypoglycaemia, headache, dehydration, electrolyte imbalance, postoperative nausea and vomiting (PONV), and increased insulin resistance [2–4]. Insulin resistance is an important factor in postoperative outcome and it is an important predictor of the length of hospital stay for patients undergoing elective surgery [5–7]. Studies have shown that paediatric patients aged 6 months to 6 years after prolonged preoperative fasting for 10-16 hours are more prone to hypotension during general anaesthesia [8–11].

Studies have demonstrated that adhering to the minimum fasting recommendation does not significantly affect the stomach volume and pH to increase the risk of regurgitation and aspiration. In effect, prolonged preoperative fasting is not beneficial rather it may be associated with complications (1-4). Literature revealed several possible reasons for excessive preoperative fasting such as patients′ lack of knowledge regarding the rationale for preoperative fasting, rapidly changing surgical schedules, the absence of a formal fasting policy or staff members′ inadequate knowledge of the policy, general mistrust on the part of practitioners that patients will understand and comply with a fasting policy [12]. At Princess Marina Hospital (PMH), Gaborone, Botswana, the investigators believed that the practice of preoperative fasting period is unnecessarily prolonged from the midnight on the day of surgery without considering the time of the procedure to be done. The objectives of this study are to assess preoperative fasting period and compare it with the ASA recommendation, to assess patients’ fasting duration for foods and liquids prior to the surgery, to investigate preoperative fasting instructions and evaluate other potential factors that may influence the patients’ fasting period.

## Methods

After permission was obtained from three institutional ethical review boards, University of Botswana, Botswana Ministry of Health and Princess Marina Hospital from June 5 to September 3, 2015, the study was conducted in Princess Marina Hospital, a referral hospital located in Gaborone, Botswana. PMH was purposefully selected for convenience. It is believed that both the patients and PMH are similar to the other health facilities around the country with regard to the study problem. This is a descriptive cross-sectional study of preoperative fasting times among elective surgical patients at the Hospital's main operating theatre. A total numbers of 260 patients were selected after explaining the research and obtain informed consent. The sample size required was calculated using the Cochran's formula for minimum sample size determination in cross-sectional study [13]. The study population consisted of elective surgical patients. Aged 5 years and above, and ASA physical status class I and class II. Exclusion criteria were age below 5 years, patients requiring emergency surgery, ASA physical status class III and above, Pregnant women, patients at increased risk of aspiration (obese, symptomatic gastro-esophageal reflux disease, hiatus hernia, achalasia, diabetes mellitus, etc), patients on intravenous fluid, parenteral or enteral tube feeding and mentally challenged persons. Convenience sampling of all patients coming to the theatre per day for elective surgical procedure and who met with the inclusion criteria for the study were assessed until a sample size of two hundred and sixty was acquired. A semi-structured interviewer administered questionnaire was used to collect data from the respondents. Patients were interviewed by investigators as they arrive at the reception area of the main operating theatre using questionnaire. The questionnaire consists of patients’ sociodemographic characteristics, fasting instructions given, time of the last meal and drink ingested and the time anaesthesia started. The questionnaire was administered on the patients who are 14 years and above but on the parents or guardian of children between 5-14 years. Data was entered into Statistical Package for Social Science (SPSS) version 23 software for analysis. Data was presented as frequency distribution. Independent sample t-test was used to test the association between time of operation and duration of fasting for liquids and solids among respondents. The level of significance was set at p < 0 .05.

## Results

A total of 260 patients were included in this study. Majority (65.7%) of the respondents were in the age group 30 years and above, with mean age of 37.5±18.6 years. More than half (55.4%) of the respondents were males while 44.6% represents the female population. More than one-tenth (19.6%) of the respondents had no formal education and 16.2% had completed the tertiary level of education. In more than a quarter (31.9%) of the respondents the type of surgery was general surgery followed by the Orthopedics surgery (28.5%). Prior to surgery, 61.9% of the patients received general anaesthesia while 38.1% received regional blocks. The details of sociodemographic characteristics of respondents and the type of surgery and anaesthesia are in Table 1. The scheduled times of surgery was not noted in all lists; but the estimated time of each surgery would take was written. Fasting instructions were delivered to all patients included in the study. The fasting instructions were delivered by ward nurses for 148 (56.9%) patients and by doctors for 112 (43.1%) patients. The respondents were also asked to describe the fasting instruction they were given. Majority (98.1%) were instructed to abstain from both liquids and solids from midnight. The remaining patients (1.9%) were delivered other fasting instructions such as no eating or drinking after supper. Princess Marina Hospital provides supper for inpatients around 5:00 pm. Explanation about the importance of preoperative fasting was not delivered in majority of patients (94.2%). Fifteen patients (5.8%) reported being told why it was important to fast preoperatively, whereas the others said no explanation was given. Among those patients who reported being told why it was important to fast preoperatively, two patients (13%) didn't understand the explanation about the importance of preoperative fasting. In 168 (64.6%) patients, anaesthesia was started before midday (12:00), the rest after midday. The durations of preoperative fasting for solids and liquids are noted in Table 2. The preoperative fasting times for solids and liquids in those patients who were operated before midday were compared to those after midday. Significantly higher mean duration of liquid fasting (17.1±2.0) was found among respondents that were operated after midday compared to those that were operated before midday (14.3±1.8), p<0.001. Likewise, higher mean duration (17.9±2.1) of solid fasting was found among respondents that were operated after midday compared to those operated before midday (14.8±1.9), p<0.001 (Table 3).

**Table 1:** Sociodemographic characteristics, type of anaesthesia and surgery among elective surgical patients at princess marina hospital main operating theatre

Variables	Frequency n =260	Percentage
**Age**		
<10	20	7.7
10-19	29	11.2
20-29	40	15.4
30-39	64	24.6
40-49	43	16.5
>macr;50	64	24.6
Mean±SD	37.5±18.6	
Median (Range)	35(5-87)	
**Gender**		
Male	144	55.4
Female	116	44.6
**Highest level of Education**		
None	52	19.6
Primary	86	33.1
Secondary	81	31.2
Tertiary	42	16.2
**Type of Anaesthesia**		
General Anaesthesia	161	61.9
Regional Anaesthesia	99	38.1
**Type of Surgery**		
General surgery	83	31.9
Orthopedics Surgery	74	28.5
Pediatrics Surgery	16	6.2
Neurosurgery	11	4.2
Urology	12	4.6
ENT	10	3.8
Dental Surgery	32	12.3
Plastic Surgery	18	6.9
Other	4	1.6

**Table 2:** Preoperative fasting times among elective surgical patients at princess marina hospital main operating theatre

Variable	Mean	Median	SD	Minimum	Maximum
Fasting times for solids (hrs)	15.91	15.67	2.52	12.00	25.33
Fasting times for liquids (hrs)	15.29	14.83	2.30	12.00	22.00

**Table 3:** Association between time of operation and duration of fasting for liquids and solids among elective surgical patients at princess marina hospital main operating theatre

	Mean±SD	T	p-value		95% CI
**Time of operation**				Lower	Upper
Before Midday (liquids)	14.3±1.8	-11.646	<0.001*	-3.302	-2.347
After midday (liquids)	17.1±2.0				
Before Midday (solids)	14.8±1.9	-12.331	<0.001*	-3.725	-2.699
After midday (solids)	17.9±2.1				

* Independent sample t-test

## Discussion

This is the first survey conducted to assess the preoperative fasting times in our centre in Botswana. There is no established preoperative fasting policy in the Hospital where the study was conducted. This study showed that the vast majority of patients (98.1%) were given the traditional nil per oral (NPO) after midnight instructions for both liquids and solids, whether they are listed for early or late procedure. The obvious result of using a single order- NPO- after midnight –for a variety of surgical procedure times is that patients scheduled for late operation will have to fast for longer periods. This was shown in our study by the significant difference of the mean duration of fasting for liquids and solids among respondents who were operated after midday compared to those operated before midday. The fasting instructions were the same for liquids and solids, even though liquids have a more rapid gastric emptying time [1, 14] The study showed that the estimated duration of each operation is mentioned in all of the surgical lists. However, the scheduled time of surgery is not indicated in all lists. This makes it difficult coordinating preoperative fasting instruction with the schedule time. The importance of preoperative fasting was not explained for the vast proportion (94.2%) of patients and this probably affected adherence to the instruction. Studies have shown that patient education about the importance of preoperative fasting improves adherence to fasting instruction [4, 15, 16]. Patients may also feel that fasting for longer period than recommended is useful for them, when the reverse is correct.

All patients in our study have fasted for both liquid and solid in excess of the recommended preoperative fasting time by ASA guidelines which recommends a minimum fasting time of two hours for clear liquids and six hours for solids. The study showed that the mean fasting time is 7.65 times for clear liquids and 2.5 times for solids longer than the recommended preoperative fasting times of American Society of Anesthesiologists (ASA). In this study, the lengthy fasting times for liquids and solids when compare with ASA Guidelines, are similar to the findings in other studies on preoperative fasting times [12, 17, 18]. Prolonged period of fasting may have an adverse consequences for the patient such as headache, thirst, hunger, dehydration, electrolyte imbalance, distress, confusion, hypoglycemia, postoperative nausea and vomiting (PONV), and increased insulin resistance [2–7, 19]. Preoperative symptoms such as headache, nausea, thirst and hunger are related to the duration of fasting from food and liquid. Patients experienced more thirsty and hungry as the duration of fasting lasted beyond the recommended time [17, 7]. Prolonged pre-operative fasting increase the incidence of PONV [4, 19]. Consumption of fluid up to 2 hours prior to GA reduces postoperative vomiting [3, 20]. Children who have experience prolonged fasting are more prone to hypotension during general anaesthesia [8–11]. Prolonged fasting triggers a metabolic response that precipitates gluconeogenesis and increases the organic response to trauma. Insulin resistance is an important factor in postoperative outcome and it is an important predictor of the length of hospital stay for patients undergoing elective surgery [5–7]. Prolonged preoperative fasting in abdominal surgery results in a marked increase of insulin resistance. Abbreviation of the period of preoperative fasting diminishes insulin resistance and the organic response to trauma [21–23]. Based on our findings, we recommend that the Hospital should establish preoperative fasting policies and teach the staffs that should ensure compliance with the guideline on regular basis. Research has shown a significant reduction in fasting times for liquids and solids following putting into practice the preoperative fasting protocol [7]. The scheduled time of each surgery should be mentioned in the surgical lists in order to coordinate the fasting instruction with the time of the proposed operation. Nurses and doctors should check the list before delivering fasting instruction which should be harmonized with the time of the procedure. Patients who are to be operated after midday (12:00) should be allowed to eat light meal in the morning six hours before the time of the procedure and drink clear liquids two hours before the time of the planned procedure. Explaining the reason for preoperative fasting is useful for the patients to act in accordance with fasting instruction.

## Conclusion

This study found preoperative fasting times longer than the ASA recommendation. There is presently no preoperative fasting policy in our Hospital and a single fasting instruction is given which disallows intake of both liquids and solids after midnight. The preoperative fasting policy should clearly address the recommended fasting time for liquid and solid. The time of proposed surgery should be checked before administration of fasting instruction as follows, patients for morning list (before 12.00) should be allowed solid meals 6 hours and clear liquid 2 hours before the time of planned procedure and those scheduled for afternoon list (after 12.00) should be allowed to light meal (eg; toast with clear liquid) in the morning 6 hours and clear fluids up to 2 hours before the proposed surgery. Nurses and doctors should discourage the traditional nil per oral (NPO) after midnight and work together to ensure that instructions are consistent with ASA guidelines and that the patients understand these directives.

### What is known about this topic

- Fasting before anaesthesia reduces the risk of regurgitation and aspiration of gastric contents.
- Prolonged periods of fasting may cause complications.

### What this study adds

- This study informs that institutions should develop fasting policies and teach staffs that should ensure compliance with the guidelines on a regular basis.
- This study recommends that nurses and doctors should check surgical lists before delivering preoperative fasting instructions.
